# Gel-immersion peroral cholangioscopy-guided biopsy can maintain a clear endoscopic view during bleeding

**DOI:** 10.1055/a-2791-4888

**Published:** 2026-02-24

**Authors:** Takeshi Ogura, Kimi Bessho, Junichi Nakamura, Nga Nguyen Trong, Hiroki Nishikawa

**Affiliations:** 1 38588 Pancreatobiliary Advanced Medical Center, Osaka Medical and Pharmaceutical University Hospital, Osaka, Japan; 2 38588 Endoscopy Center, Osaka Medical and Pharmaceutical University Hospital, Osaka, Japan; 3 13010 2nd Department of Internal Medicine, Osaka Medical and Pharmaceutical University, Osaka, Japan; 4 Department of Gastroenterology, Trong Nam Cancer Hospital, Hanoi, Vietnam

The gold standard technique for the diagnosis of biliary disease is brush cytology or forceps biopsy under endoscopic retrograde cholangiopancreatography (ERCP) guidance. According to a meta-analysis of brush cytology and forceps biopsy under ERCP guidance 1 , the diagnostic yield of these techniques may not be sufficient in clinical practice. In contrast, peroral cholangioscopy (POCS) may have several advantages, such as providing direct ductal visualization and enabling targeted biopsy under visual guidance. However, bleeding from the tumor after the first biopsy can decrease visibility and make a second biopsy challenging. Water can be injected to obtain a clear endoscopic view; however, an excessive amount of injected water can cause reflux cholangitis. Recently, the use of gel immersion endoscopy using a transparent gel (Viscoclear; Otsuka Pharmaceutical Factory, Tokushima, Japan), which is more viscous than saline, has been reported to secure the visual field 2 3 4 . Technical tips for gel-immersion cholangioscopy-guided biopsy for bile duct tumors are presented.

**Fig. 1:**
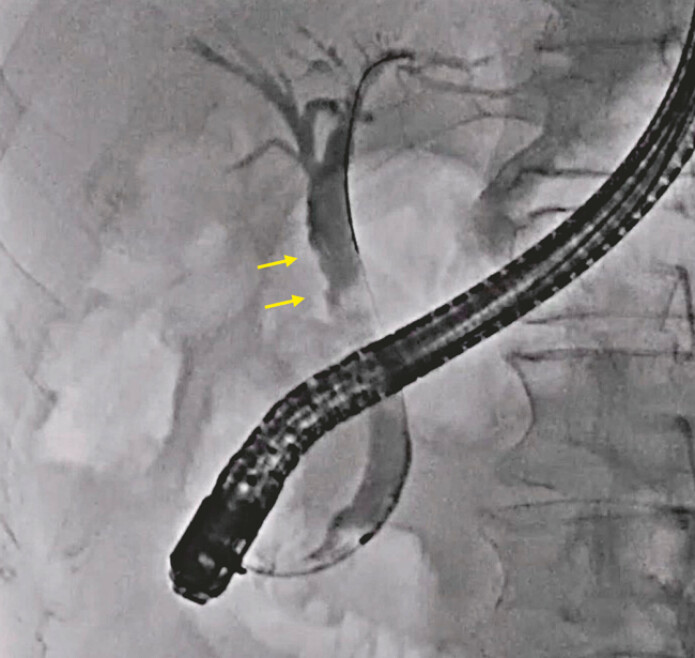
A bile duct tumor is suspected on cholangiography (arrow).

**Fig. 2:**
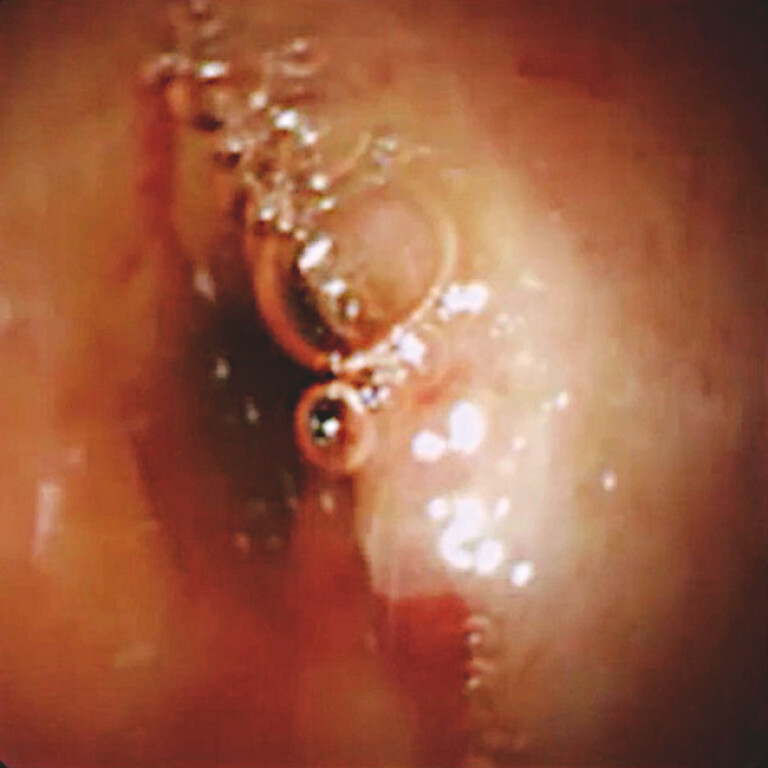
A bile duct tumor with oozing is observed on cholangioscopy.

**Fig. 3:**
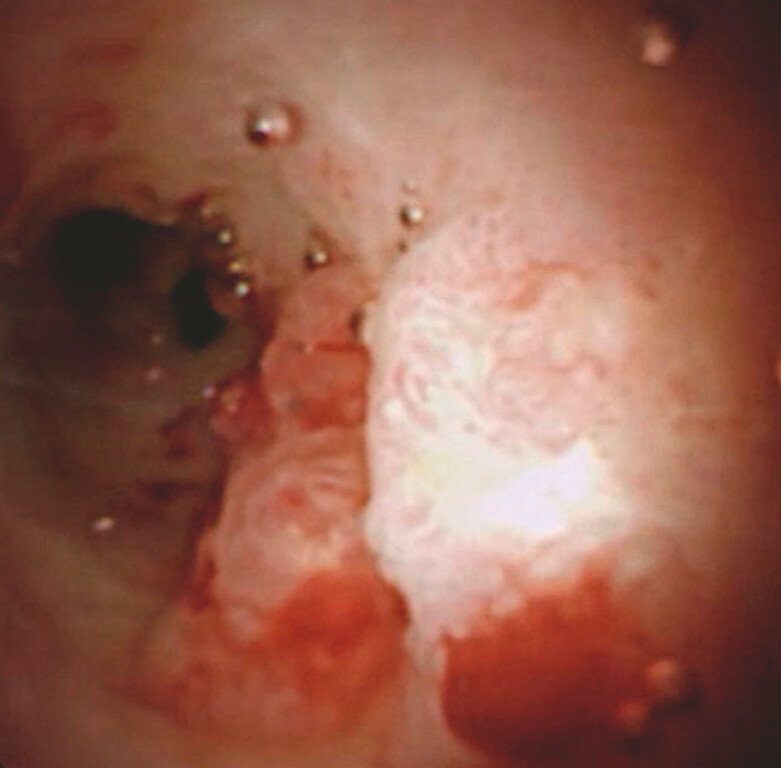
After gel injection, the tumor can be clearly observed.

**Fig. 4:**
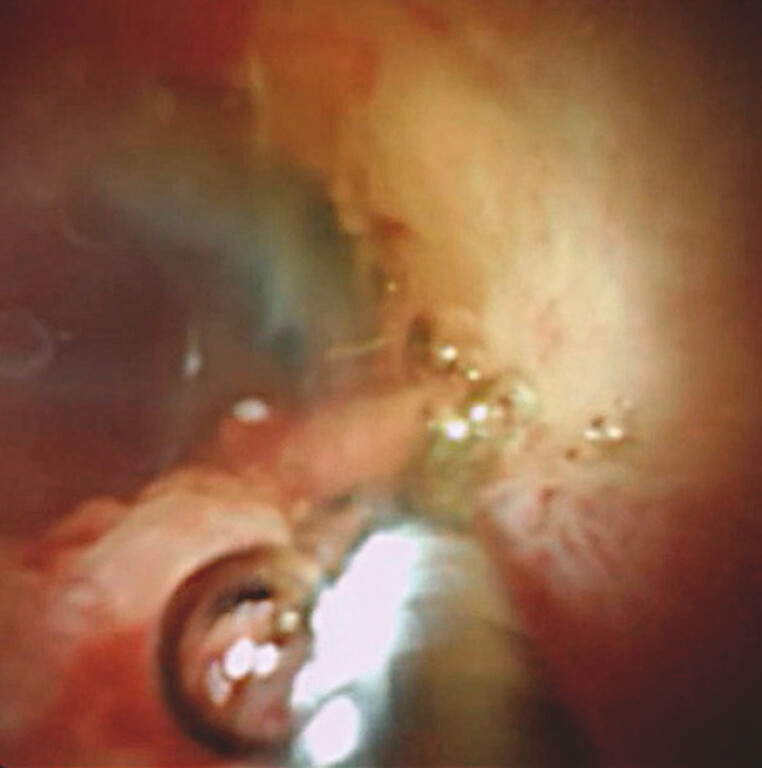
A forceps biopsy is performed.

**Fig. 5:**
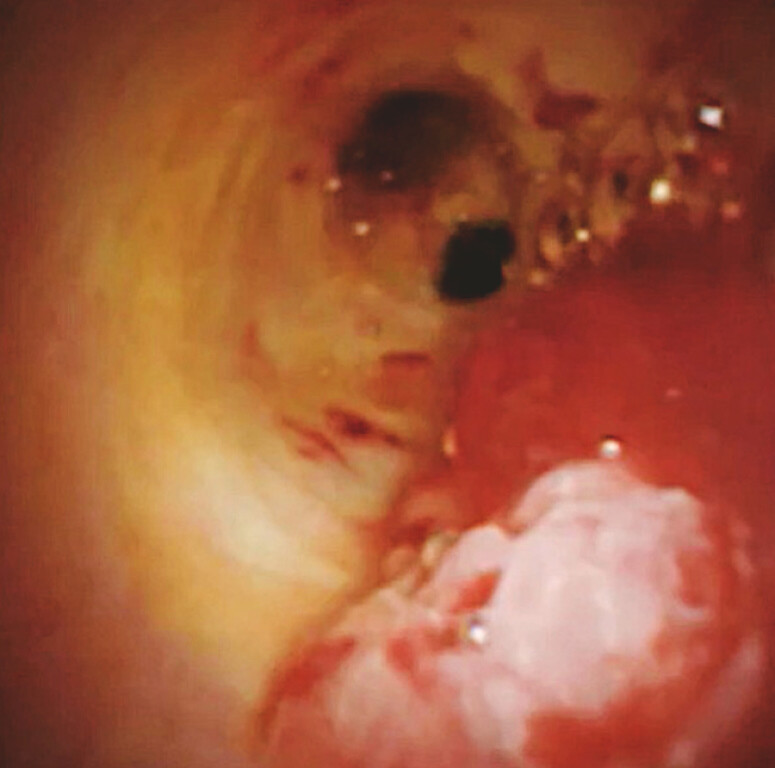
Although bleeding from the tumor has occurred, the endoscopic view is still clearly maintained.

A 71-year-old man was admitted to our hospital due to a bile duct lesion. To obtain a diagnosis, ERCP was attempted. After successful biliary cannulation, a bile duct tumor was suspected on cholangiography ( Fig. 1 ). Because no stricture of the tumor site was observed, POCS-guided biopsy was attempted. After POCS insertion, the tumor was identified ( Fig. 2 ). Because oozing from the tumor, which might suggest easy bleeding after biopsy, was observed, gel injection was first attempted. Gel injection facilitated a clear view of the tumor ( Fig. 3 ). Subsequently, target biopsy using a dedicated biopsy device was performed ( Fig. 4 ). Post-biopsy bleeding was observed. Under usual circumstances, blood would obscure the visual field, but, owing to the injected gel, a clear endoscopic view was maintained ( Fig. 5 ). The second biopsy was successfully performed without any adverse events ( Video 1 ). The gel was delivered through the working channel of the cholangioscope using a 5-mL syringe. Because the viscosity of the gel decreases when warmed to approximately body temperature, the risk of gel-induced obstructive cholangitis after endoscopic sphincterotomy is considered low. This histological diagnosis in this case was cholangiocarcinoma.

Gel-immersion peroral cholangioscopy-guided biopsy can maintain a clear endoscopic view during bleeding.Video 1

In conclusion, gel-immersion POCS-guided biopsy can be helpful for obtaining an adequate biopsy by maintaining a clear endoscopic view during bleeding.

Endoscopy_UCTN_Code_TTT_1AR_2AL Endoscopy_UCTN_Code_CCL_1AZ_2AC
